# Can treefrog phylogeographical clades and species’ phylogenetic topologies be recovered by bioacoustical analyses?

**DOI:** 10.1371/journal.pone.0169911

**Published:** 2017-02-24

**Authors:** Lucas Rodriguez Forti, Rodrigo Lingnau, Lais Carvalho Encarnação, Jaime Bertoluci, Luís Felipe Toledo

**Affiliations:** 1 Laboratório Multiusuário de Bioacústica (LMBio) e Laboratório de História Natural de Anfíbios Brasileiros (LaHNAB), Departamento de Biologia Animal, Instituto de Biologia, Universidade Estadual de Campinas, Campinas, São Paulo, Brazil; 2 Universidade Tecnológica Federal do Paraná, Francisco Beltrão, Paraná, Brazil; 3 Programa de Pós-Graduação em Diversidade Animal, Instituto de Biologia, Universidade Federal da Bahia, Campus Universitário de Ondina, Salvador, Bahia, Brazil; 4 Departamento de Ciências Biológicas, Escola Superior de Agricultura Luiz de Queiroz, Universidade de São Paulo, Piracicaba, São Paulo, Brazil; National Cheng Kung University, TAIWAN

## Abstract

Phenotypic traits, such as the frog advertisement call, are generally correlated with interspecific genetic variation, and, as a consequence of strong sexual selection, these behaviors may carry a phylogenetic signal. However, variation in acoustic traits is not always correlated with genetic differences between populations (intraspecific variation); phenotypic plasticity and environmental variables may explain part of such variation. For example, local processes can affect acoustic properties in different lineages due to differences in physical structure, climatic conditions, and biotic interactions, particularly when populations are isolated. However, acoustic traits can be used to test phylogenetic hypotheses. We analyzed the advertisement calls of *Dendropsophus elegans* males from 18 sites and compared them with those of four closely related congeneric species, in order to test for differences between inter and intraspecific variation. We analyzed 451 calls of 45 males of these five species. Because males from distant sites were grouped together without population congruence, differences found in advertisement calls among individuals were not correlated with phylogeographical clades. Phylogenetic and cluster analyses of the *D*. *elegans* clades and those of closely related species grouped all five species into the same topology, as reported by previous molecular and morphological phylogenies. However, the topology of the *D*. *elegans* phylogeographical clades did not match the topology previously reported. Acoustic communication in *D*. *elegans* seems to be conserved among populations, and the phylogeographical history of the species does not explain the variation among lineages in call properties, despite some congruent phylogenetic signals evident at the species level. Based on molecular clocks retrieved from the literature, it seems that more than 6.5 million years of divergence (late Miocene) are necessary to allow significant changes to occur in the acoustic properties of these treefrog calls, making it possible to recover their phylogenetic history only based on acoustic evidence.

## Introduction

Acoustic communication is crucial during the anuran life cycle, and is used by males of many species for mate attraction, territory defense, and predator avoidance [1, 2]. Among the types of calls anurans emit (see [3]), the advertisement call is unique in that it is subject to sexual selection [4–6]. Therefore, it contains phylogenetic information that may be useful in evolutionary studies. Such phylogenetic signals are generally detected when comparing closely related species, or higher taxa, of the same clade [7–9]. Spectral properties, such as dominant frequency peaks, usually exhibit low intraspecific variation, and therefore are the main acoustic traits used for distinguishing between species [4, 10].

The acoustic information that is encoded in the advertisement call can be influenced by intrinsic factors, such as body size, or extrinsic factors, such as environmental temperature, chorus density, or social context [3, 5, 11, 12]. These factors vary among locations, so call variation among populations should also exist. Indeed, previous studies have shown that geographical variation in advertisement calls could result in divergence and consequent speciation [13–15]. In this context, as for any species with populations that are limited by geographical barriers, the variation in acoustic properties of different populations may be strongly influenced by genetic features [16]. The fact that most anurans do not disperse over long distances [17–19] supports this hypothesis.

Although some studies have found a positive relationship between genetic and acoustic distances among populations [20, 21], others have found that geographical variation in sexual signals does not covary with genetic distances among populations [19, 22, 23]. In some cases, it has even been suggested that phenotypic (call) evolution could be decoupled from genotypic features [19], but many acoustic properties of the advertisement calls of anurans are more influenced by extrinsic factors than by genetic determination [5, 11, 12]. Therefore, it remains unclear in what circumstances phylogeographical history can be inferred from acoustic similarities among conspecific populations.

*Dendropsophus elegans* is a Neotropical treefrog, and one of almost 100 species currently described in the genus [24]. It was first described from the municipality of Caravelas, Bahia state, Brazil, and is widely distributed in the Brazilian Atlantic forest, occurring from the state of Santa Catarina to Bahia [24]. The advertisement call of *D*. *elegans* was first described by Bastos and Haddad [25], based on five individuals recorded at Ubatuba, São Paulo state. It was described as a pulsed sound with a duration of less than 0.2 s. The frequency spectrum ranged from 2 to 5 kHz, and the dominant frequency was between 3 and 4 kHz [25]. No information is available on variation in the advertisement call among populations. Nevertheless, recent molecular analyses have revealed a clear phylogeographical structure, with three main clades within this species that are structured along a latitudinal gradient [26]. These authors (Tonini et al.) reported high genetic diversity among populations, and provided a hypothesis for *D*. *elegans* biogeography based on the mitochondrial gene *ND2* [26].

Therefore, we analyzed the advertisement calls of *D*. *elegans* clades and those of closely related species, in order to test whether it is possible to predict evolutionary relationships based on acoustic data, both at the intra- and interspecific levels. Considering genetic information that is related to calling behavior in anurans [27, 28], we hypothesized that the genetic lineages would support differences in acoustic traits for topological reconstruction.

## Materials and methods

### Species, sites, and procedures

**Fig 1:**
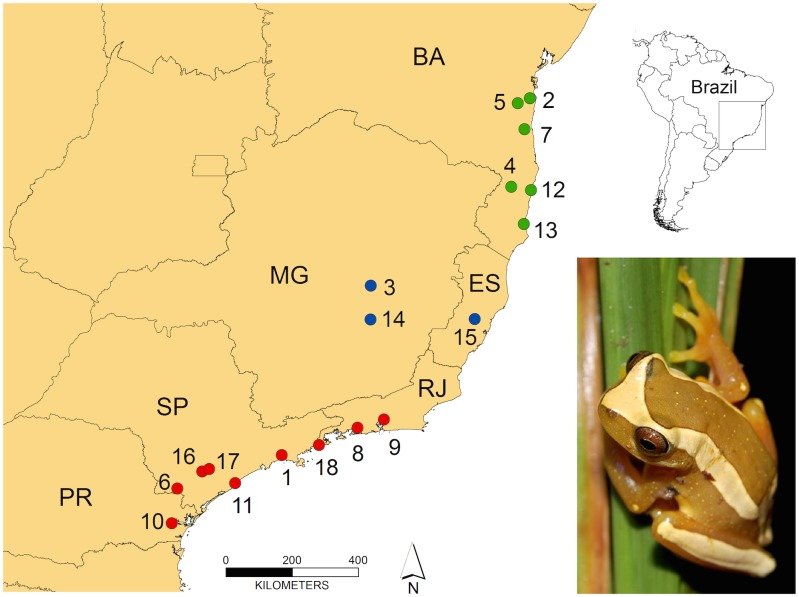
Sampling locations of *Dendropsophus elegans*. The colors of the dots correspond to each individual’s phylogeographical clade (as in [26]): northern (green), central (blue), and southern (red). The location numbers are provided in S1 Table.

We compared male advertisement calls from three distinct phylogeographical clades. The samples comprised 37 individuals from 18 locations across *D*. *elegans*’ distribution (Fig 1). In addition, we obtained advertisement calls of four congeneric species (all close to *D*. *elegans*, according to Duellman et al. [29]): *D*. *bipunctatus*, *D*. *ebraccatus*, *D*. *leucophyllatus*, and *D*. *triangulum*. Advertisement calls were recorded using the following recorders: Uher 4000, Marantz PMD660, Marantz Cassette Recorder PMD222, Tascam Portable DAT Recorder DAP1, Tascam DR 07, Sony Cassette recorder MZ-D55, and Sony DAT TCD-D100; and microphones: Sennheiser ME 66, Yoga HT 81, and Sony ECM. Recordings obtained by us and others donated by colleagues (mentioned in the acknowledgments) were digitized and saved as “WAV” files. All audio files are available in the Fonoteca Neotropical Jacques Vielliard (FNJV 12836–38, 13051, 30752–57, 31844, 32985, 32990, 33000, 33001, 33003, 32991, 33022–30, 33033, and 33035–39) and in public repository (https://zenodo.org).

We estimated geographical distances between locations using Google Earth. We obtained the mean temperature of the warmest quarter from each location by extracting data from worldclim/bioclim [30] using ArcMap 10.1 [31]. All of the rasters were generated at a scale of 1 km.

### Sound analyses

We used Raven Pro 64 1.4 (Cornell Laboratory of Ornithology) for the acoustic analyses. Different spectrogram configurations were used, depending on the analysis. For spectral data, we used a Fast Fourier Transformation (FFT) of 1024. For temporal data, we used a FFT of 256. For both analyses, the window overlap was 50%. From 451 calls of 45 males, we measured 12 acoustic properties (Table 1). Spectral measurements were obtained by selecting four functions in the source “choose measurements” in Raven: (1) Frequency 5% (Hz) and (2) Frequency 95% (Hz)—these two measures included maximum frequency and minimum frequency, ignoring 5% below and above the total energy in the selected call; (3) Bandwidth 90% (Hz)—frequency range that included 90% of the energy distribution (difference between frequencies 95% and 5%); and (4) Max Frequency (Hz)—peak of dominant frequency (the frequency at which the power is maximum within the call). For temporal properties, we made precise selections of calls in the spectrogram, and visually counted the pulses. Amplitude and frequency modulation were measured independently in the first and second half of the call. Amplitude modulation was measured using the Raven function “Peak Power (dB)”.

**Table 1:** Acoustic properties analyzed, character codes, and state delimitation for phylogenetic analysis.

**Temporal properties**
Pulse number per call (A1): 4–11.8 (0); 11.9–19.8 (1); 19.9–27.7 (2); more than 27.7 (3)
Call duration (s) (A2): 0.049–0.114 (0); 0.115–0.180 (1); 0.181–0.240 (2); more than 0.240 (3)
Pulse rate (A3): 74–96.3 (0); 96.4–118.7 (1); 118.8–141.1 (2); more than 141.1 (3)
First note duration (s) (A4): 0.031–0.075 (0); 0.076–0.121 (1); 0.122–0.166 (2); more than 0.166 (3)
Note rate (notes per minute) (A5): 9–48 (0); 49–84 (1); more than 84 (2)
**Mechanistic properties**
Note composition (M1): single note (0); multi-note (1)
Amplitude modulation (dB) (M2): (-5)–(-2.8) (0); (-2.7)–(-0.5) (1); (-0.4)–1.8 (2); more than 1.8 (3)
**Spectral properties**
Range frequency (Hz) (F1): 462–705 (0); 706–948 (1); 949–1192 (2); more than 1192 (3)
Maximum frequency (Hz) (F2): 2668–3530 (0); 3531–4392 (1); 4393–4636 (2); more than 4636 (3)
Minimum frequency (Hz) (F3): 2206–3151 (0); 3151–4096 (1); more than 4096 (2)
Peak of dominant frequency (Hz) (F4): 2451–3470 (0); 3471–4489 (1); more than 4489 (2)
Frequency modulation (Hz) (F5): (-78)–41 (0); 42–160 (1); 161–279 (2); more than 279 (3)

Characters numbers (A1–5; M1–2; F1–5) refer to the columns in the character matrix (see S1 Table).

### Statistical analyses

We conducted a principal component analysis (PCA) to test whether we could predict phylogeographical clades using acoustic property variation among *D*. *elegans* individuals. We used correlation matrices with a bootstrap number of 1000, and in order to test the congruence between acoustic features, geography, and phylogenetic data, we constructed two similarity trees using all of the quantitative acoustic properties on two levels: (1) comparing *D*. *elegans* individuals, and (2) comparing *D*. *elegans* sites and those of congeneric species. We used cluster analysis based on the Bray-Curtis index of similarity through the unweighted pair group method with arithmetic mean grouping model. We also performed 1000 randomizations to estimate bootstrap values for all of the joinings (see [32]). We performed a pairwise correlation analysis among the acoustic traits and the mean temperature of the warmest quarter to investigate possible effects of climatic conditions on the acoustic traits. Statistical analyses were conducted using Past 2.17 [33] and Statistica 7.1 [34].

### Phylogenetic analyses

We elaborated a matrix for the phylogenetic analyses that codified the acoustic properties into characters inside three acoustic modalities: (1) temporal, (2) mechanistic, and (3) spectral. We delimitated states for both categorical and numerical acoustic properties, and used two states for categorical variables [absence (0) and presence (1)]. We transformed numerical variables into categorical states based on range and presence of outliers, creating three states when outliers were observed and four states when they were not present. Our codification and state delimitation are detailed in Table 1. We elaborated our matrix in the software NDE (Nexus Data Editor), and exported a readable file for the software TNT (Tree analysis using New Technology) [35], in which we ran a maximum parsimony analysis. We generated the best 100 trees using Tree Bisection Reconnection (TBR), with *D*. *bipunctatus* as the outgroup. Subsequently, we selected the best tree in terms of species topology and population phylogeographical clades, and resampled with 10,000 replicates using a standard bootstrap procedure. Values at nodes represented absolute frequencies and frequency differences (GC, Group present/Contradicted).

### Divergence time estimations

**Fig 2:**
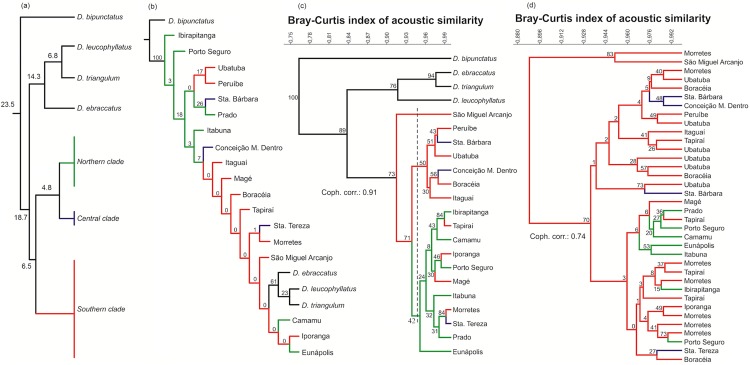
Different arrangements of species and populations of *Dendropsophus*. (a) Phylogenetic representation of phylogeographical clades of *Dendropsophus elegans* (as in [26]) and closely related species (as in [29]). Numbers at nodes are estimates of the divergence time. (b) Phylogenetic tree based on acoustic data using the maximum parsimony analysis method. Numbers at nodes are values resulting from a standard bootstrap resampling. (c) Dendrogram with four outgroup *Dendropsophus* species and 18 *D*. *elegans* populations resulting from a hierarchical cluster analysis based on similarities in call properties (dashed vertical line on the three main branches). (d) Dendrogram of 37 *D*. *elegans* individuals resulting from a hierarchical cluster analysis based on similarities in acoustic properties. In both dendrograms, the numbers at nodes are values obtained from standard bootstrap resampling. The colors of the branches correspond to each individual’s phylogeographical clade (as in [26]): northern (green), central (blue), and southern (red).

We used the divergence time estimations of Tonini et al. (2013) (phylogeographical perspective) and Duellman et al. (2016) (for species topology) in order to establish the temporal gradient of evolutionary changes in our phylogenetic representation (Fig 2).

## Results

**Fig 3:**
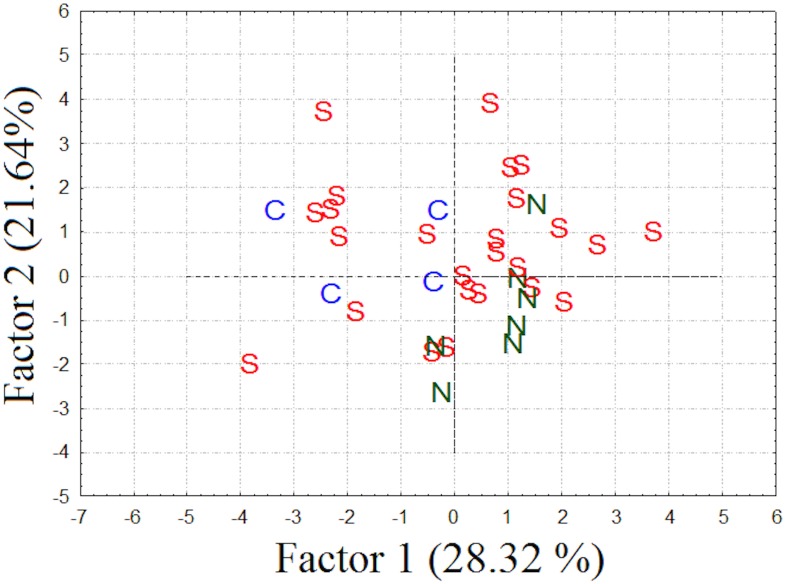
Distribution of individual scores related to phylogeographical clades along the first and second principal components using 12 acoustic properties of the advertisement call of *Dendropsophus elegans*. The colors and letters correspond to each individual’s phylogeographical clade (as in [26]): N, northern (green); C, central (blue); and S, southern (red).

The advertisement call of *D*. *elegans* was composed of two notes that were separated by a short interval, which varied from 12 to 43 ms (n = 37). The first note had multiple pulses (range, 7–44; n = 37), while the second only comprised one or two very fused pulses. The peak power was usually (76%) in the second note. Both the frequency and amplitude changed across the call, but the extent to which this occurred varied between individuals (range, 47–400 Hz and 0.03–11 dB, respectively). The variation in call properties among individuals was not due to geographical differences, so we could not predict the males’ phylogeographical positions using acoustic evidence (see the PCA results in Fig 3 and Table 2). In addition, the cluster analysis of the 37 *D*. *elegans* males organized them without population congruence (Fig 2).

**Table 2:** Results of a principal component analysis of the general structure of acoustic variables in *Dendropsophus elegans*.

Variable	Factor 1	Factor 2	Factor 3	Factor 4
Peak of dominant frequency	-0.8700	0.2004	-0.2400	-0.1079
Call duration	0.4280	0.6366	-0.5677	-0.1119
First note duration	0.6281	0.4296	-0.2105	-0.1782
Note rate	-0.0532	0.4002	-0.1426	0.7151
Range frequency	0.1042	0.3196	0.7601	-0.4567
Maximum frequency	-0.8264	0.4089	0.2204	-0.1855
Minimum Frequency	-0.8571	0.1442	-0.3658	0.1704
Pulse number	0.3813	0.7582	0.2253	0.3330
Pulse rate	-0.1274	0.1774	0.7991	0.4246
Frequency modulation	-0.3864	0.7051	0.0285	-0.2592
Amplitude modulation	0.1973	0.4434	-0.1262	-0.1456
Eigenvalue	3.1156	2.3805	1.9107	1.2188
% variance	28.3238	21.6407	17.3696	11.0802
Cumulative Eigenvalue	3.1156	5.4961	7.4068	8.6256
Cumulative %	28.3238	49.9645	67.3341	78.4143

**Fig 4:**
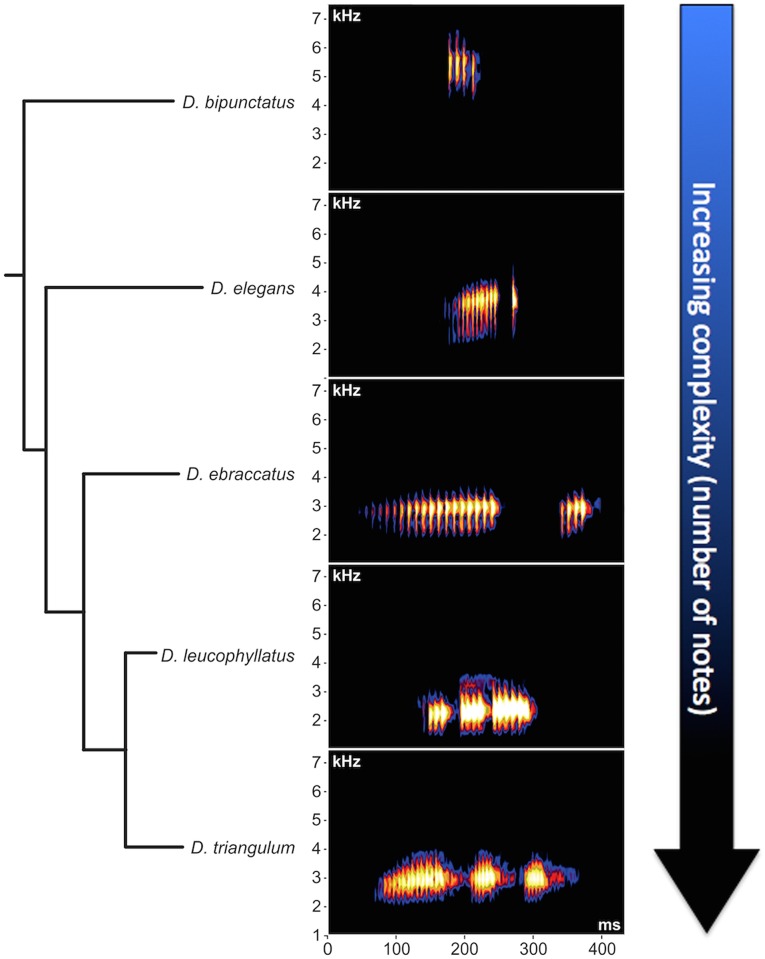
Phylogenetic representation of the spectrograms of five treefrog species. The phylogenetic topology of these species is based on [29], and the figure is arranged in a manner that highlights increasing advertisement call complexity (in terms of number of notes).

General acoustic properties, such as frequency modulation and call composition with pulsed notes, were similar among some of the congeneric species that were phylogenetically closely related to *D*. *elegans* (Fig 4). A detailed comparison of the acoustic properties of *D*. *elegans* with those of other species from a geographical perspective is presented in S2 Table.

Despite low bootstrap values at some nodes, our phylogenetic analysis was successful in recovering the species topology (as in [29]), but not the phylogeographical clades of *D*. *elegans* as described by Tonini et al. [26] (Fig 2). Some populations of *D*. *elegans* represented a sister group for the branch formed by *D*. *ebraccatus*, *D*. *leucophyllatus*, and *D*. *triangulum*. We obtained a similar result in the cluster analysis, which grouped other species’ and *D*. *elegans* populations according to acoustic similarity (Fig 2). However, we noted some differences regarding the phylogenetic tree, as *D*. *leucophyllatus* changed position with *D*. *ebraccatus* and *D*. *elegans* populations were clustered into an exclusive branch. The populations were structured into three major clades (see the dashed vertical line in Fig 2c), without geographical congruence. The following distant populations were grouped in both phylogenetic and phenetic trees: Iporanga and Eunápolis (1300 km straight-line distance), Iporanga and Porto Seguro (1345 km straight-line distance), Tapiraí and Ibirapitanga (1385 km straight-line distance), and Morretes and Porto Seguro (1425 km straight-line distance).

We did not find any correlation among the acoustic traits and summer mean temperature in the locations studied (Table 3).

**Table 3:** Results of a pairwise correlation analysis of acoustic variables in *Dendropsophus elegans* and the mean temperature of the warmest quarter (summer) in the locations studied (n = 18).

Variable	Correlation coefficient (r)	*p* value
Peak of dominant frequency	-0.0609	0.81
Call duration	-0.2327	0.35
First note duration	-0.1859	0.46
Note rate	0.004	0.98
Range frequency	-0.3333	0.17
Maximum frequency	-0.3005	0.22
Minimum Frequency	-0.0674	0.79
Pulse number	-0.1843	0.46
Pulse rate	0.0876	0.37
Frequency modulation	-0.373	0.12
Amplitude modulation	-0.3202	0.19

## Discussion

The use of acoustic traits has been avoided in amphibian systematics, mainly because acoustic traits consist of several continuous characters and a few discrete ones, and because there is an assumption that calls could substantially be influenced by extrinsic factors such as environmental and social conditions. Despite the fact that morphological data are also influenced by extrinsic factors, and have many continuous characters [36], morphology is still commonly used. We argue that advertisement calls can provide discrete, categorical data for parsimony analyses, and are able to corroborate molecular and even total evidence phylogenies. Our findings, although based on a small sample size, corroborate the current molecular topology of *D*. *elegans* congenerics. Our results show congruence between acoustic evidence and species topology (see also [7–9]), thus confirming that the study of anuran vocalizations helps in fine-tuning their systematics. It is possible that some acoustic traits exhibit high levels of homoplasy, resulting in an accurate phylogenetic arrangement of species [30, 37, 38]. Sexual selection probably maintains the consistency and regularity of these sexual signals by stabilizing selection, which has been observed in other species (for example [39]).

However, the acoustic similarity among individuals and lineages of *D*. *elegans* failed to group individuals within their respective populations, or populations according to their geographical regions. Despite genetic differentiation among the three regions, the acoustic evidence suggests that the phylogeographical clades do not have different calls (see [26]). However, this lack of geographical consistency demonstrates that call evolution has a rate of divergence that is probably distinct from that of population genetics. Acoustic attributes are multidimensional phenotypic traits that exhibit a plasticity that is decoupled from population genotypic features [19]. Moreover, the tropical environment can be more complex than temperate habitats, and its ecological structure, such as the background noise produced by the calls of many other syntopic species, may influence the calls of target species [40]. Therefore, at the same site, we would expect variation in call properties among males from different reproductive environments.

Several Neotropical studies have shown that geographical variation in sexual signals does not covary with genetic distance [19, 22, 23, 39], which may be a consequence of random evolution or drift [32]. In contrast, concordance between genetic and acoustic features has been shown among populations, both in the Neotropics [20, 21] and in temperate Europe [41]. Therefore, acoustic similarities could be used as a tool to investigate relationships among species, but not among phylogeographical clades.

*Dendropsophus elegans* has a two-note advertisement call, in which notes are separated by a short interval. The phylogenetic arrangement suggests a gradual change in the number of notes between species (Fig 4). Based on this, simple calls (containing one note) are probably the plesiomorphic condition, which was followed by an increasing number of notes. Therefore, complex calls (more than one note) should be the apomorphic state of this character.

Although calls are innate and genetically determined in frogs [27, 28, 42], many acoustic properties may show variation and plasticity [12, 43]. Distinctive background noise and the physical structure of the habitat may cause important changes in calling behavior and in other acoustic properties, resulting in divergence [44]. In this context, each population of *D*. *elegans*, connected or not by gene flow, may exhibit adjustments according to their habitat conditions; these adjustments may be mediated by phenotypic plasticity or genetic change. If plasticity drives changes in call traits, no geographical or genetic distance will correlate with phenotype. However, even when including individuals in a cluster analysis, males were not arranged according to a geographical region or population, indicating that acoustic communication in *D*. *elegans* is conserved in its distribution. In addition, acoustic variation was not correlated with air temperature at the different sites. Therefore, we suggest that call property variation is related to other local predictors. Despite the genetic structure of *D*. *elegans* populations [26], they are acoustically connected (see also [45]).

In conclusion, the phylogenetic signal in anurans’ advertisement call (as a phenotypic trait) can be identified when higher taxonomic levels are compared as different sister species, and possibly among distinct genera. However, on a smaller scale, such as when comparing calls among phylogeographical clades, the variation in some call properties, which have been modeled by specific environmental conditions, may strongly influence anuran advertisement calls locally, such as background noise and the physical characteristics of different habitats. Therefore, based on our results, we suggest that large geographical distances between populations are not always sufficient to acoustically segregate them. Based on the divergence time estimations of Tonini et al. (2013), we suggest that more than 6.5 million years would be necessary for the diversification of acoustic traits. That timeframe would enable us to find congruence of bioacoustical analyses with phylogenetic hypotheses, as we found at the species level, but not at the phylogeographical clade level. Our study investigated general patterns using data from the literature; future studies should include larger sample sizes, and combine genetic and acoustic data from the same individuals, which would generate further accurate findings.

## Supporting information

S1 Table Data matrix of characters from the acoustic coding (A1–A5), mechanistic coding (M1–M2), and spectral coding (F1–F5) of advertisement calls of 18 populations of *Dendropsophus elegans* and four outgroup-related species. (DOCX)

S2 Table Acoustic properties (mean ± SD, range) of 18 populations of *Dendropsophus elegans* and four closely related species as outgroups. (DOCX)
