# Supplementary material for: Can treefrog phylogeographical clades and species’ phylogenetic topologies be recovered by bioacoustical analyses?
Source: PLoS One. 2017 Feb 24;12(2):e0169911. doi: 10.1371/journal.pone.0169911 (PMC5325193; doi:10.1371/journal.pone.0169911)
Supplement: S1 Table — (DOCX) [file pone.0169911.s001.docx]

Supporting information

**S1 Table.** Data matrix of characters from the acoustic coding (A1–A5), mechanistic coding (M1–M2), and spectral coding (F1-F5) of advertisement calls of 18 populations of *Dendropsophus elegans* and four outgroup-related species.

| **Site/species** | **A1** | **A2** | **A3** | **A4** | **A5** | **A6** | **M1** | **M2** | **F1** | **F2** | **F3** | **F4** | **F5** |
| --- | --- | --- | --- | --- | --- | --- | --- | --- | --- | --- | --- | --- | --- |
| 1. Boraceia | 2 | 1 | 2 | 1 | 0 | 1 | 1 | 3 | 1 | 1 | 1 | 1 | 3 |
| 1. Camamu | 1 | 1 | 0 | 1 | 0 | 0 | 1 | 3 | 2 | 1 | 0 | 0 | 1 |
| 1. Conceição do Mato Dentro | 1 | 1 | 1 | 1 | 0 | 0 | 1 | 2 | 2 | 1 | 1 | 1 | 3 |
| 1. Eunápolis | 1 | 1 | 0 | 0 | 0 | 2 | 1 | 2 | 1 | 1 | 0 | 0 | 3 |
| 1. Ibirapitinga | 0 | 0 | 1 | 0 | 0 | 0 | 1 | 1 | 2 | 1 | 0 | 1 | 1 |
| 1. Iporanga | 2 | 1 | 3 | 0 | 0 | 0 | 1 | 3 | 1 | 1 | 0 | 0 | 1 |
| 1. Itabuna | 1 | 0 | 3 | 1 | 0 | 2 | 1 | 2 | 1 | 1 | 0 | 1 | 3 |
| 1. Itaguaí | 2 | 1 | 1 | 1 | 0 | 0 | 1 | 2 | 2 | 1 | 0 | 1 | 3 |
| 1. Magé | 2 | 1 | 1 | 1 | 0 | 0 | 1 | 2 | 1 | 1 | 0 | 0 | 2 |
| 1. Morretes | 1 | 1 | 2 | 1 | 0 | 0 | 1 | 3 | 1 | 1 | 0 | 1 | 1 |
| 1. Peruíbe | 1 | 0 | 1 | 0 | 0 | 1 | 1 | 3 | 2 | 2 | 1 | 1 | 3 |
| 1. Porto Seguro | 0 | 0 | 1 | 0 | 0 | 0 | 1 | 0 | 1 | 1 | 0 | 1 | 1 |
| 1. Prado | 1 | 0 | 2 | 0 | 0 | 0 | 1 | 1 | 1 | 1 | 1 | 1 | 0 |
| 1. Santa Bárbara | 1 | 0 | 2 | 0 | 0 | 0 | 1 | 1 | 1 | 1 | 1 | 1 | 3 |
| 1. Santa Tereza | 1 | 1 | 2 | 1 | 0 | 0 | 1 | 0 | 1 | 1 | 0 | 1 | 1 |
| 1. São Miguel Arcanjo | 1 | 1 | 2 | 1 | 0 | 0 | 1 | 3 | 3 | 1 | 0 | 0 | 2 |
| 1. Tapiraí | 1 | 1 | 1 | 1 | 0 | 0 | 1 | 3 | 2 | 1 | 0 | 1 | 1 |
| 1. Ubatuba | 1 | 0 | 3 | 0 | 1 | 0 | 1 | 2 | 2 | 2 | 1 | 1 | 2 |
| *D. ebraccatus* | 2 | 3 | 0 | 3 | 0 | 0 | 1 | 3 | 0 | 0 | 0 | 0 | 0 |
| *D. triangulum* | 3 | 3 | 1 | 2 | 0 | 0 | 1 | 1 | 0 | 0 | 0 | 0 | 0 |
| *D. leucophyllatus* | 1 | 2 | 1 | 0 | 0 | 2 | 1 | 1 | 0 | 0 | 0 | 0 | 0 |
| *D. bipunctatus* | 0 | 0 | 1 | 0 | 2 | 0 | 0 | 2 | 2 | 3 | 2 | 2 | 3 |
