# Supplementary material for: Can treefrog phylogeographical clades and species’ phylogenetic topologies be recovered by bioacoustical analyses?
Source: PLoS One. 2017 Feb 24;12(2):e0169911. doi: 10.1371/journal.pone.0169911 (PMC5325193; doi:10.1371/journal.pone.0169911)
Supplement: S2 Table — (DOCX) [file pone.0169911.s002.docx]

**Supporting information**

**S2 Table.** Acoustic properties (mean ± SD, range) of 18 populations of *Dendropsophus elegans* and four closely related species as outgroups.

| **Group** | **Phylogeographical clade** | **Population (number of calls; number of males)** | **Range frequency (Hz)** | **Minimum frequency (Hz)** | **Peak of dominant frequency (Hz)** | **Maximum frequency (Hz)** | **Call duration (s)** | **Pulses per call** | **Pulse rate (p/s)** | **First note duration (s)** | **Note rate (notes per minute)** | **Frequency modulation (Hz)** | **Amplitude modulation (dB)** | **Number of notes** |
| --- | --- | --- | --- | --- | --- | --- | --- | --- | --- | --- | --- | --- | --- | --- |
| *Dendropsophus elegans* | Southern | Boraceia  (47; 3) | 856 ± 175 (646–1453) | 3284 ± 122 (3100–3609) | 3738 ± 211 (3359–4125) | 4140 ± 226 (3962–4968) | 0.178 ± 0.037 (0.108–0.249) | 20 ± 8  (12–46) | 123 ± 59 (58–240) | 0.097 ± 0.019 (0.065–0.121) | 59 ± 37 (21–96) | 400 ± 317 (129–750) | 4.3 ± 0.6 (3.9–5) | 2 |
|  | Northern | Camamu  (14; 1) | 972 ± 98 (818–1119) | 2900 ± 32 (2842–2928) | 3365 ± 53 (3273–3445) | 3872 ± 78 (3746–3962) | 0.129 ± 0.014 (0.105–0.143) | 11 ± 1.4 (10–14) | 89 ± 10 (76–114) | 0.08 ± 0.02 (0.05–0.105) | 30 | 86 | 2.1 | 2 |
|  | Central | Conceição do Mato Dentro  (7; 1) | 1033 ± 237 (818–1550) | 3168 ± 240 (2627–3316) | 3746 ± 223 (3402–3919) | 4202 ± 78 (4048–4263) | 0.135 ± 0.004 (0.129–0.142) | 15 ± 4.4 (14–26) | 117 ± 37 (98–201) | 0.08 ± 0.004 (0.08–0.09) | 17 | 366 | 0.9 | 2 |
|  | Northern | Eunápolis  (9; 1) | 802 ± 120 (656–1031) | 2833 ± 94 (2625–2953) | 3302 ± 110 (3140–3515) | 3635 ± 62 (3562–3750) | 0.147 ± 0.014 (0.133–0.180) | 12 ± 1.4 (10–15) | 84 ± 14 (66–109) | 0.05 ± 0.02 (0.03–0.07) | 87 | 304 | 0.1 | 2 |
|  | Northern | Ibirapitanga  (5; 1) | 990 ± 86 (861–1076) | 2919 ± 77 (2799–2971) | 3677 ± 103 (3574–3789) | 3910 ± 150 (3660–4048) | 0.093 ± 0.007 (0.082–0.101) | 10 ± 1  (9–12) | 109 ± 7 (99–120) | 0.05 ± 0.007 (0.04–0.05) | 12 | 151 | -1.1 | 2 |
|  | Southern | Iporanga  (20; 1) | 857 ± 112 (656–1031) | 3110 ± 55 (3046–3187) | 3473 ± 94 (3281–3609) | 3967 ± 98 (3796–4078) | 0.109 ± 0.006 (0.093–0.123) | 16 ± 1.4 (13–19) | 147 ± 11 (130–176) | 0.06 ± 0.008 (0.06–0.08) | 9 | 47 | 2.6 | 2 |
|  | Northern | Itabuna  (8; 1) | 937 ± 253 (562–1359) | 2818 ± 223 (2390–3000) | 3468 ± 130 (3281–3703) | 3755 ± 12 (3562–3937) | 0.135 ± 0.022 (0.099–0.168) | 19 ± 6.4 (12–27) | 142 ± 40 (86–200) | 0.08 ± 0.006 (0.07–0.09) | 80 | 305 | 1.4 | 2 |
|  | Southern | Itaguaí  (13; 1) | 1189 ± 311 (750–1500) | 2956 ± 339 (2625–3468) | 3858 ± 182 (3281–4031) | 4146 ± 56 (4031–4218) | 0.162 ± 0.043 (0.122–0.252) | 18 ± 6.3 (13–32) | 112 ± 9 (102–127) | 0.116 ± 0.03 (0.08–0.169) | 20 | 281 | 0.7 | 2 |
|  | Southern | Magé  (20; 1) | 738 ± 190 (515–984) | 3035 ± 173 (2765–3234) | 3449 ± 99 (3281–3562) | 3773 ± 47 (3703–3890) | 0.162 ± 0.009 (0.139–0.186) | 17 ± 1.9 (14–21) | 106 ± 9 (88–127) | 0.107 ± 0.01 (0.08–0.117) | 48 | 164 | -0.3 | 2 |
|  | Southern | Morretes  (47; 7) | 874 ± 207 (624–1615) | 3082 ± 219 (2325–3337) | 3585 ± 298 (2347–4005) | 3957 ± 112 (3768–4177) | 0.124 ± 0.032 (0.091–0.202) | 15 ± 3.3 (11–25) | 129 ± 33 (82–252) | 0.07 ± 0.01 (0.06–0.104) | 32 ± 15 (19–64) | 100 ±81 (-11–205) | 2.1 ± 4.7 (-3.4–11.1) | 2 |
|  | Southern | Peruíbe  (2; 1) | 1007 ± 33 (984–1031) | 3398 ± 33 (3375–3422) | 3797 ± 0 (3797) | 4406 ± 66 (4359–4453) | 0.104 ± 0.006 (0.100–0.109) | 12 ± 1.4 (11–13) | 115 ± 20 (100–130) | 0.05 ± 0.005 (0.05–0.06) | 84 | 281 | 2 | 2 |
|  | Northern | Porto Seguro (16; 2) | 794 ± 73 (689–904) | 2941 ± 137 (2713–3143) | 3482 ± 201 (3014–3832) | 3736 ± 148 (3488–3876) | 0.109 ± 0.033 (0.062–0.149) | 10 ± 1.6 (8–13) | 101 ± 26 (65–145) | 0.07 ± 0.009 (0.06–0.09) | 11 ± 12 (2–20) | 43.5 ± 122 (-43–130) | -3.4 ± 3.6 (-6–-0.8) | 2 |
|  | Northern | Prado  (15; 2) | 781 ± 256 (515–1265) | 3199 ± 348 (2765–3562) | 3634 ± 196 (3328–3890) | 3981 ± 160 (3750–4171) | 0.104 ± 0.016 (0.074–0.125) | 14 ± 4.1 (11–25) | 141 ± 53 (90–297) | 0.06 ± 0.01 (0.05–0.08) | 11* (M = 1) | -11 ± 49 (-46–24) | -0.55 ± 1.6 (-1.7–0.6) | 2 |
|  | Central | Santa Bárbara  (22; 2) | 907 ± 217 (516–1765) | 3471 ± 234 (3122–3768) | 3997 ± 256 (3488–4478) | 4379 ± 110 (4220–5015) | 0.097 ± 0.016 (0.083–0.252) | 11 ± 2.8 (8–46) | 119 ± 23 (88–389) | 0.05 ± 0.008 (0.04–0.07) | 68 ± 31 (46–90) | 376 ± 45 (344–409) | -1.5 ± 4.4 (-4.7–1.6) | 2 |
|  | Central | Santa Tereza  (10; 1) | 781 ± 167 (646–1205) | 3406 ± 130 (3057–3509) | 3632 ± 200 (3100–3854) | 4188 ± 77 (4069–4349) | 0.137 ± 0.018 (0.104–0.164) | 15 ± 4  (10–23) | 105 ± 18 (84–140) | 0.08 ± 0.01 (0.05–0.108) | 15 | 86 | -4.6 | 2 |
|  | Southern | São Miguel Arcanjo  (13; 1) | 1434 ± 222 (1248–2110) | 2507 ± 88 (2368–2627) | 3084 ± 87 (2928–3273) | 3942 ± 197 (3789–4565) | 0.119 ± 0.002 (0.114–0.123) | 15 ± 0.7 (14–16) | 127 ± 6 (113–135) | 0.08 ± 0.009 (0.07–0.104) | 18 | 194 | 3.9 | 2 |
|  | Southern | Tapiraí  (55; 4) | 1030 ± 272 (560–1765) | 2914 ± 193 (2411–3230) | 3574 ± 131 (3140–3789) | 3945 ± 162 (3656–4435) | 0.125 ± 0.017 (0.083–0.183) | 14 ± 2  (9–22) | 112 ± 9 (88–134) | 0.09 ± 0.01 (0.05–0.104) | 16 ± 2 (13–18) | 123 ± 144 (-65–258) | 3.1 ± 2.9 (0.9–7.2) | 2 |
|  | Southern | Ubatuba  (55; 6) | 1071 ± 237 (656–1734) | 3320 ± 225 (2812–3703) | 3964 ± 188 (3515–4265) | 4393 ± 214 (4031–5015) | 0.082 ± 0.016 (0.05–0.121) | 13 ± 4.7 (7–30) | 163 ± 60 (106–389) | 0.05 ± 0.01 (0.04–0.08) | 24 ± 15 (7–48) | 195 ± 165 (-47–421) | (-0.03) ±2.99 (-3.9–4.3) | 2 |
| Outgroup | | *Dendropsophus ebraccatus*  (8; 1) | 640 ± 48 (559–689) | 2417 ± 15 (2411–2454) | 2906 ± 32 (2885–2971) | 3057 ± 39 (3014–3100) | 0.270 ± 0.067 (0.195–0.361) | 19 ± 3.6 (17–26) | 74 ± 7 (65–87) | 0.211 ± 0.004 (0.208–0.216) | 15 | 64 | 4.2 | 2 |
|  |  | *Dendropsophus triangulum*  (19; 3) | 501 ± 108 (344 -646) | 2543 ± 263 (2110–2859) | 2915 ± 277 (2670–3328) | 3043 ± 297 (2756–3421) | 0.345 ± 0.1 (0.242–0.495) | 31 ± 9  (15–43) | 91 ± 23 (61–127) | 0.114 ± 0.049 (0.046–0.186) | 24.4 ± 9.5 (11–30) | 74 ± 109 (0–375) | 2.6 ± 3.4 (0.5–12.4) | 2–3 |
|  |  | *Dendropsophus leucophyllatus* (47; 3) | 461 ± 123 (328–609) | 2206 ± 46 (2109–2297) | 2451 ± 77 (2343–2625) | 2668 ± 115 (2531–2859) | 0.182 ± 0.04 (0.145–0.300) | 16.2 ± 4.6 (11–26) | 88 ± 12 (68–113) | 0.031 ± 0.025 (0.015–0.160) | 114 ± 33 (91–138) | 77.9 ± 79 (0–239) | 2.1 ± 1.28 (0.5–4.6) | 2–3 |
|  |  | *Dendropsophus bipunctatus*  (12; 1) | 1074 ± 432 (796–2250) | 5042 ± 227 (4640–5484) | 5507 ± 198 (5109–5812) | 6117 ± 358 (5625–6890) | 0.048 ± 0.010 (0.031–0.065) | 4 ± 1.2  (2–6) | 83 ± 11 (64–102) | 0.048 ± 0.010 (0.031–0.065) | 10.8 | 281 | 2.4 | 1 |
